# Association of Environmental Temperature and Relative Humidity with Ocular and Flank Temperatures in Dromedary Camels

**DOI:** 10.3390/ani15030309

**Published:** 2025-01-22

**Authors:** Asim Faraz, Naod Thomas Masebo, Syeda Maryam Hussain, Abdul Waheed, Hafiz Muhammad Ishaq, Nasir Ali Tauqir, Ali Raza Abbasi, Faizan Saleem, Barbara Padalino

**Affiliations:** 1 Department of Livestock and Poultry Production, Bahauddin Zakariya University, Multan 60800, Pakistan; drasimfaraz@bzu.edu.pk (A.F.); drabdulwaheed@bzu.edu.pk (A.W.); dr.ishaq@bzu.edu.pk (H.M.I.); mrfs2632@gmail.com (F.S.); 2 Department of Agricultural and Food Sciences, University of Bologna, 40127 Bologna, Italy; naodthomas.masebo2@unibo.it; 3 Department of Livestock Production and Management, Pir Mehr Ali Shah—Arid Agriculture University, Rawalpindi 46300, Pakistan; syedamaryam@uaar.edu.pk; 4 Department of Animal Nutrition, The Islamia University of Bahawalpur, Punjab 63100, Pakistan; tauqir041@hotmail.com; 5 Faculty of Veterinary Science, Muhammad Nawaz Shareef University of Agriculture, Multan 66000, Pakistan; alirazaabbasi1965@gmail.com; 6 Faculty of Science and Engineering, Southern Cross University, Lismore, NSW 2480, Australia

**Keywords:** thermography, dromedary camels, physiology, thermal stress, selective brain cooling

## Abstract

Heat stress represents significant challenges for livestock, adversely affecting their production, reproduction, and overall welfare. This study aimed to explore the interrelationships between environmental and animal-related factors and the flank temperature (FT) and eye temperature (ET) recorded using IRT in dromedary camels. This study was conducted in the Cholistan Desert in 2023, and IRT images of the eyes and flanks were captured from 510 camels across 54 herds. During the image analyses, pictures taken from 499 camels were of good quality and included. The camels were of both sexes and of various ages (minimum 3 years, pubertal and adult stages), and they had diverse physiological statuses (breeding, immature, lactating, non-lactating, and pregnant). Before taking the IRT pictures, ambient temperature and humidity were registered using a weather station, and light intensity was recorded using a lux meter. The ET was associated only with physiological status (*p* < 0.05), with pregnant females showing the lowest values, while no effects of physiological status, sex, or age were found for FT. The environmental temperature showed a positive correlation with both ET (r = 0.7887) and FT (r = 0.6280), highlighting the sensitivity of camel thermoregulation to temperature fluctuations. As expected, a strong positive correlation between ET and FT (r = 0.6643) was found. Conversely, a significant negative correlation was observed between humidity and ET (−0.7444) and FT (−0.5519), indicating that higher humidity levels lead to decreased temperatures in both regions. Light intensity (lux) exhibited minimal influence on both temperatures, with correlations of 0.1019 for ET and 0.2650 for FT. This study contributes to the field of precision livestock farming by suggesting a possible application of IRT for detecting thermal stress in camels in pastoral settings.

## Simple Summary

New approaches focusing on non-invasive techniques for measuring animals’ body temperatures are very important from the perspective of animal welfare. Dromedary camels possess a wider thermo-neutral zone due to their morpho-physiological adaptability. We aimed to measure the superficial skin temperature of camels’ flanks and eyes using infra-red thermography and its link with environmental and animal-related factors. A total of 510 dromedary camels of both sexes with different physiological states and ages were assessed. While the camels’ flank temperatures were not affected by age, sex, or physiological status, their eye temperatures were significantly associated with their physiological status, with pregnant camels showing the lowest value. The results showed that eye temperature and flank temperature were associated with environmental temperature and humidity (*p* < 0.001) but not with light intensity. Notably, a 10% increase in humidity resulted in a decrease of approximately 0.727 °C in eye and 1.564 °C in flank temperature, reflecting a more pronounced response of the flank temperature. Our findings suggest that camel’s eye and flank temperatures recorded using infra-red thermography could be a useful indicator of thermal stress in dromedary camels. Their response is different from other animals, likely due to the unique physiological factors of this species. Further studies are needed to confirm whether infra-red thermography could help to identify camels at risk of heat stress, allowing for timely management interventions.

## 1. Introduction

Infra-red thermography (IRT) is an innovative, non-invasive technology that has gained prominence in monitoring health conditions and stress in farm animals. IRT is cost and time effective, reliable and can repeatably determine animals’ early disease and habits. It is also useful for thermal stress and thermal characterization [1,2], animal welfare, production [3], reproduction [4,5], and detecting population sizes [6]. Radiometric images are created by evaluating an animal’s body surface temperature in correlation with the environment [7]. Therefore, IRT is a feasible tool for improving animal health monitoring by evaluating their thermal stress levels [8,9]. It is used remotely without handling, restricting or sedating the animals, or compromising the animals’ welfare, thus significantly reducing any extra stress caused to the animals. Heat stress and causative factors are quite easily and accurately monitored using IRT due to an increase in the animals’ skin temperature [9].

Assessing animals’ thermal comfort is important within a welfare protocol [10,11,12]. Exposure to severe environmental thermal stress for a prolonged time not only reduces animals’ production but also threatens their welfare [11] and disturbs their health [12,13]. Thermal heat stress negatively impacts animals’ physiology and productivity, as their heat dissipation capacity is exceeded due to internal blood flow [13,14]. Stress-induced hyperthermia changes the physiological responses and the sympathetic and parasympathetic activities of animals [15] due to the heat carried by the blood capillaries to the adjacent tissues and skin. The heat generated due to higher temperatures is transmitted to the skin by the increased capillary blood flow and is dissipated as infrared energy, which and then be measured using IRT [16]. Normally, healthy animals’ bodies have a uniform distribution of temperature, but sometimes, some anatomical parts are warmer due to stress and thermal deregulation [17]. This increase in the animals’ surface temperature may be due to either direct radiative heat absorption from the ambient or from the blood circulation from visceral organs throughout various body parts during the temperature variations during the day [18,19].

Dromedaries are adapted due to their unique anatomical, physiological, and behavioral characteristics to face severe thermal stress and long periods of dehydration [20,21]. The comparative genomic and transcriptomic analyses of dromedaries have shown various features with high potential for desert adaptation [22,23], such as enhanced metabolism for fats, energy, and salt (sodium re-absorption), water storage, and osmoregulation. A camel’s flank temperature is relatively close to its core body temperature [17] but is highly influenced by the environmental temperature. However, it is easily maintained by the potential thermal driving power of more sweat glands, a higher sweating rate, and higher blood flow [7].

Camels can experience hyperthermia, with body temperatures (Tb) reaching as high as 41 °C during the day. This physiological adaptation helps them to reduce the temperature gradient between their body and the environment, thereby minimizing heat gain [24]. For heat dissipation, camels have two mechanisms: adaptive heterothermy or thermo-conformity [25] to keep their core temperature between 36 and 38 °C. Due to these mechanisms, dromedary camels can thrive in harsh climates by allowing their body temperature (Tb) to fluctuate (often exceeding 6 °C) through various physiological and behavioral strategies [26]. All this allows them to minimize water loss through evaporative cooling by reducing sweating [27]. The role of the hypothalamus in balancing these physiological mechanisms with thermoregulation is complex. The hypothalamus, particularly the suprachiasmatic nucleus, regulates these thermoregulatory processes by integrating signals from both internal and external environments, thereby orchestrating daily rhythms in body temperature that align with circadian cycles [28]. When faced with high ambient temperatures (Ta) and low humidity, the hypothalamus triggers physiological responses such as vasodilation and increased respiratory rates to enhance heat dissipation [29]. Additionally, dehydration influences food intake and modifies the timing of the camels’ daily rhythms, leading to potential internal desynchronization to avoid further complications due to thermoregulation [30]. This intricate interplay and unique combination of adaptations between external conditions and hypothalamic function underscores camels’ remarkable ability to adapt to extreme environments and underscores their evolutionary success. Further research into these mechanisms will enhance our understanding of camel physiology and its implications for livestock management in similar climates.

Sweat glands are distributed throughout camel skin over nearly the entire body except for the lips, external nares, and perianal region [31] to remove heat through water vapor. Meanwhile, their thick fur (up to 4 inches) traps these glands to avoid water loss. Interestingly, dromedary camels always sit in a recumbent position by facing the sun to facilitate air flow under their bodies, dissipating heat and avoiding full-body exposure to the heat [32]. Dehydrated camels exhibit an expanded range of body temperatures from 34 to 41 °C, which serves as a physiological adaptation to minimize further water loss [33]. Camel eyes have two thick upper and lower eyelids and a nictitating membrane guarding the eye, along with a fat layer in their facial pocket. The third eyelid has the most receptors in the sensory capsule, which are important for eye protection and have significant immunologic functions [34]. The fat cells around the ocular orbit keep the eyes protected from hard situations. A camel’s ocular orbits and brain are richly supplied with arteries and veins [35], while the spiral vasoconstriction in the carotid rete helps in “selective brain cooling”. The arterial blood suffusing the brain enters the carotid rete at the base of hypothalamus, which acts as a heat exchanger for preventing brain damage [36,37,38]. The blood entering the brain has a ~0.5 °C lower temperature comparatively [39,40], and upon a rise in the ocular temperature, this heat is dissipated by the rich arteries and higher blood flow immediately due to their critical link and location with the brain.

IRT has emerged as a valuable non-invasive tool for assessing thermal responses in a diverse range of wildlife species to monitor physiological parameters like heart rate and respiration rate [41]. It can correlate body surface temperatures with environmental conditions, providing insights into thermal stress, reproductive status, and metabolic rates in different species [42,43]. The regions of the body targeted for performing IRT differ between species, as different degrees of correlation with RT and ambient temperature have been observed [44]. Studies have shown that camels’ flank temperatures are cooler than their eye temperatures during heat stress. The higher temperatures in the eyes are due to increased metabolic activity related to vision and environmental awareness [26]. Research has shown that other species also exhibit thermoregulatory adaptations, but these are significantly different from camels. Cattle eyes are not adaptable for thermal regulation; hence, their temperature increase leads to higher respiration rates and sweating [45]. Therefore, cattle rely mainly on behavioral adaptations such as shade seeking and increased water consumption rather than making physiological changes. Many mammals may not have protective eyelids like camels, thus making them vulnerable to withstanding the same level of thermal regulation [46]. The uniformity of body temperature regulation in cattle and horses is critical for supporting their health and performance under varying environmental conditions. This thermoregulatory mechanism is in contrast with camels, highlighting the differences in physiological adaptations among these species [47,48]. Cattle and horses maintain stable temperatures across different anatomical sites, underscoring their resilience in fluctuating climates and during physical activity [14]. Moreover, in livestock and horses, eye temperature measured using IRT has been validated as an indicator of emotions, particularly fear and distress [15].

This study hypothesizes that ET and FT are positively associated with the environmental T and H in dromedary camels, and they may also be related to the age, sex, and physiological states of the animals. The overall objective of the current research is to describe the ET and FT range in dromedary camels of different ages, sexes, and physiological states and investigate possible associations with T and H and light intensity. Understanding these associations is the first step to determine whether IRT could be used as a tool to monitor welfare in camels.

## 2. Materials and Methods

### 2.1. Ethical Statement

This study was approved by the Department of Livestock and Poultry Production, Bahauddin Zakariya University, Multan, Pakistan Animal Ethics Committee (Protocol number DLPP/272/27 November 2022).

### 2.2. Study Location

The present study was performed in the Southern Punjab province, Pakistan, from 29 September to 7 October 2023, with a mean ambient temperature and relative humidity of 33.4 °C (min–max: 21.3–40.9 °C) and 48.1% (min–max: 20.9–88%), respectively. Fifty-four (54) dromedary camel pastoralist herds inhabiting eight different localities within the neighborhood of Multan and the Cholistan desert (Lohari Gate (n = 11), Kotla Pul (n = 10), Channan Pir (n = 9), Gelewal (n = 7), Nag Shah (n = 5), Naubahar Pul (n = 5), Gograan (n = 4), and Chak 97 (n = 3)) were visited. A total of 510 male and female dromedary camels (*Barela* and *Marecha*) with light to fawn coat color were randomly selected for this study. These camels represented different age categories (young, puberty, and adult) and physiological statuses (breeding, immature, lactating, non-lactating, and pregnant) from a total of 1050 camels present in the herds. The camels evaluated during this study were selected on the basis of good health conditions to avoid any interventions with thermo-graphic temperatures.

Local community leaders were contacted through a colleague to facilitate and ease communication with herd managers and pastoralists. Subsequently, they were approached through a proper network, ensuring their participation voluntarily. Each herd was visited only once, following their previous schedule to respect pastoralists’ routines. The visited camel herd size ranged from 5 to 63 animals, with an average size of 43 camel heads. The camel rearing practices were broadly similar among the herds selected in the specified area. The camels were permitted to roam freely and were fed in the nearby cultivated areas during the day. Sometimes, they were provided with supplemental feed sources as fodders from the agricultural fields. Milk and meat production were the main rearing and breeding purposes of all the herds involved in the present study. Dairy camels were milked twice daily (morning and afternoon), but the pictures were taken outside the milking period.

### 2.3. Thermal Imaging

**Figure 1:**
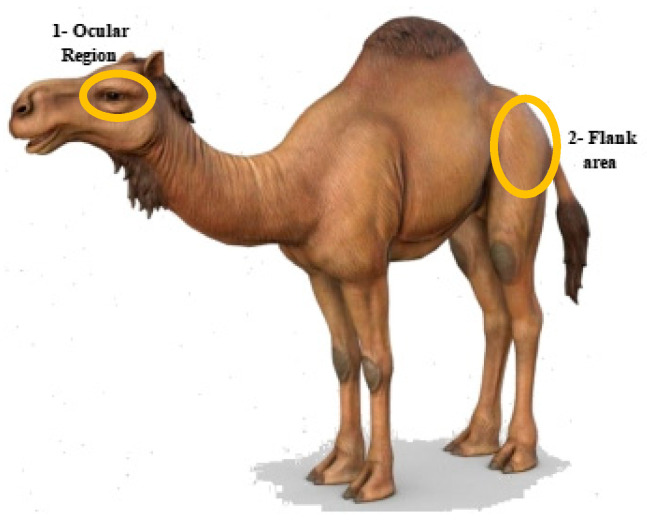
Ocular region and flank area for IRT in dromedary camels.

A thermographic camera (FLIR E76 24°; FLIR Systems AB, Danderyd, Sweden) was used for capturing images during this study. The camera was calibrated using the environmental temperature and relative humidity recorded by a weather station (Kestrel 4000, Wet Des Moines, IA, USA) before each use. This IR thermal camera has a thermal sensitivity of <0.05 °C at temperatures of ≥30 °C, having an automatic hot/cold temperature detection range between −20 °C and 1000 °C and a detector efficacy of 640 × 480 (307,200 pixels). This integrated color camera had a minimum focus distance of 15 cm, 5.0 megapixels, and it provided 4× the pixel data for creating a super-resolution image of 1280 × 960. The infrared camera had a laser pointer for focusing images and a laser distance meter to measure the distance to the specific body area measurement. Static thermal images for measuring the skin’s superficial temperature (°C) (SST) were taken at a 90° angle from a distance of 1 m [48]. To minimize the impact of human errors and environmental factors, thermo-graphic evaluations were consistently conducted in the field by the same operator (NTM). At least two pictures were taken for each camel; IRT images of either the left or the right side flank regions and either the left or right eye regions were captured to measure the superficial surface temperatures while the camels were either in a standing or recumbent position (Figure 1).

The imaging session was conducted during the day, between 7AM and 7PM. Before taking each IRT image, the environmental parameters (temperature (T) and humidity (H)) were recorded by a weather station (Kestrel 4000, Nielsen-Kellerman Company, Boothwyn, PA, USA) and noted. In addition, a digital lux meter (HoldPeak HP-881D, The Zhuhai JiDa Huapu Company, Zhuhai, China) was used to record the intensity of the light at the time of image capturing, and the lux was also noted.

Then, each picture was evaluated for quality and imported for analysis of the ET and FT using specific software (FLIR Tools^®^ 5.X) to extract the maximal temperature of the eye and the flank after having selected the specific area (this is specific tool is only available via this software). These values were then recorded in an Excel spreadsheet. However, during this process, only pictures from 499 camels were included, while the rest (n = 11) were discarded due to quality problems.

### 2.4. Statistical Analysis

Descriptive statistics were computed to summarize the data characteristics using Statistix version 8.1. Moreover, a descriptive analysis of ET and FT for age, sex, and physiological status was conducted. The fix factor effects of animal-related factors, namely age, sex, and physiological status, on ET and ST were tested using an ANOVA. Posthoc tests (i.e., Least Significant Difference (LSD) at a 5 percent significance level) were also applied in cases of significant effects.

To investigate the effects of environmental-related factors (Temperature (T (°C)), Humidity (H (%)), and lux levels) on the response variables (Flank Temperature Average (FTA) and Eye Temperature Average (ETA)), a simple linear univariate regression model was employed using R programming. The regression model was formulated as follows:

y=α+βx

where:

*y* = dependent variable
α= intercept
βx= is slope/rate of change (β) and independent variable (*x*)

Differences between means were assessed using the least significant difference test at a 5% significance level. This approach allowed for pairwise comparisons among group means to determine statistically significant differences. Bivariate correlations among the variables were computed to assess the strength and direction of relationships between pairs of variables.

The Pearson correlation coefficient was calculated using the following formula:

$$r_{xy}=\frac{\sum \left(x-\bar{x})(y-\bar{y}\right)}{\sqrt{\sum {\left(x-\bar{x}\right)}^{2}.\sum ({y-\bar{y})}^{2}}}$$

where:

$r_{xy}=$ correlation between *x* and *y*
$\sum \left(x-\bar{x}\right)=$ sum of deviation of *x* from its mean
$\sum \left(y-\bar{y}\right)=$ Ssum of deviation of *y* from its mean
$\sum {\left(x-\bar{x}\right)}^{2}=$ sum of squared deviation of *x* from its mean
$\sum ({y-\bar{y})}^{2}=$ Ssum of squared deviation of *y* from its mean

This comprehensive approach facilitated a robust analysis of how environmental parameters influence temperature averages, providing valuable insights into their interrelationships.

## 3. Results

### 3.1. Association of Eye Temperature (ET) with Age, Sex, and Physiological State

**Figure 2:**
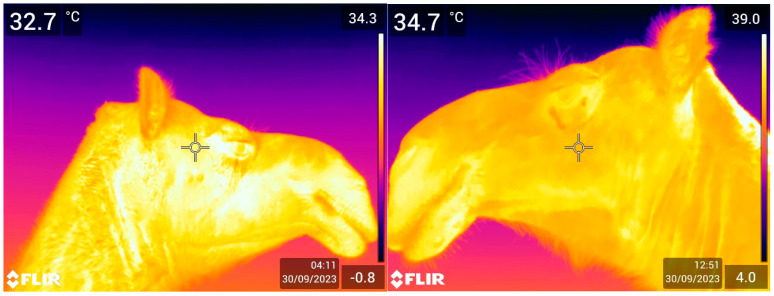
Two pictures taken of the ocular region using IRT in dromedary camels. The temperatures shown in the top corners are determined automatically by the thermo camera. The top left corner is the coldest point, and the top right corner is the hottest point of the picture. The date, time, and emissivity setting are displayed in the bottom right corner.

Table 1 shows the average eye temperature (ET) with standard errors for each category of the sampled camels. The average eye temperature varied slightly across the different age classes, although no statistically significant effects of the age classes were found. Sex also had no significant effect, and a similar average ET was observed between the male and female camels. Only the physiological status resulted in a significant effect, with pregnant camels showing a significantly lower average eye temperature (34.75 °C) compared to non-pregnant camels, both lactating and not lactating (35.2 °C and 35.3 °C, respectively). Figure 2 shows two examples of ET.

### 3.2. Association of Flank Temperature (FT) with Age and Sex

**Figure 3:**
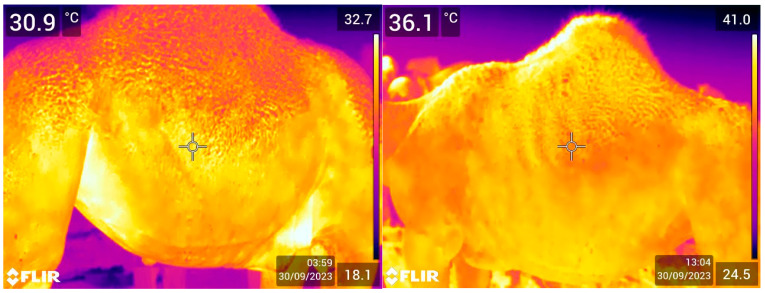
Two pictures taken of the flank region using IRT in dromedary camels. The temperatures shown in the top corners are produced automatically by the thermo camera. the top left corner is the coldest point, and the top right corner is the hottest point of the picture. The date, time, and emissivity setting are displayed in the bottom right corner.

The data indicate (Table 2) that the flank temperature in the camels remained relatively stable across different age classes, sexes, and physiological statuses, reflected in the high *p* values in each category. This suggests a robust thermoregulatory system that allows dromedary camels to maintain relatively stable body temperatures regardless of these factors. Similar to ET, the highest FT (35.32 ± 0.17) was in non-lactating camels, and the lowest (34.75 ± 0.13) was in the pregnant ones (Table 2). The recorded FT values of 36.62 °C (adults), 37.03 °C (pubertal), and 37.12 °C (young) were statistically non-significant (*p* = 0.5867). Sex-wise, the average FT values in the males (37.18 °C) and females (36.83 °C) were not significantly different (*p* = 0.7713). Based on physiological status, the average FT varied among the following groups: breeding (37.42 °C), immature (37.06 °C), lactating (36.73 °C), non-lactating (37.89 °C), and pregnant (36.31 °C), but this variation was not statistically significant. Figure 3 shows two examples of FT.

### 3.3. Association of Camels’ Superficial Skin Temperature and Environment

Table 3 shows the results of the simple linear regression and relationships between average eye temperature (ETAvg) and average flank temperature (FTAvg) and environmental temperature (T), relative humidity (H), and lux levels in dromedary camels. The ET increases by 0.3092 units for an increase in each degree of temperature (°C), while the FT increases by 0.7012 units. The FT is more sensitive to changes in humidity than ET, with a decrease of 0.1564 °C for FT compared to 0.0727 °C for ET per 1% increase in humidity. Temperature had a significant positive relationship with both ET and FT in the dromedary camels. The data indicate that approximately 64% of the ET variation was mainly due to the environmental temperature, while its effects on flank temperature were observed to be only 43%. Therefore, the ET is more sensitive to the increase in temperature. Both ET and FT showed moderate multi-collinearity with environmental temperature and humidity, as indicated by VIF values of around 4.4. the effects of humidity on the ocular region were observed to be 49%, while the flank noted to be 30%, both showing a moderate negative relationship. The lux level had negligible effects on ET and FT, indicated by their low R^2^ values.

### 3.4. Correlation Between Temperature and Environment

The correlations presented in Table 4 provide insights into the relationships between the physiological temperatures of the dromedary camels and their environmental factors including humidity and temperature. A strong positive correlation was found between T and ET (0.7887) and T and FT (0.6280) due to effects of ambient temperature on the camels’ bodies. Similarly, a strong positive correlation (0.6643) existed between ET and FT: as one temperature rose, the other increased simultaneously. The ambient humidity had a strong inverse relation with the environmental temperature (−0.8785), and as the ambient temperature rose, the RH tended to decrease significantly. Ambient humidity had a strong negative relationship with ET (−0.7444) and a moderate negative relationship with FT (−0.5519). The lux effect was more noticeable in the flank area, with a correlation of 0.265, and in the eyes, it was minimal, at 0.1019.

## 4. Discussion

This study documented the eye temperature (ET) and flank temperature (FT) ranges of dromedary camels of different ages, sexes, and physiological states kept under pastoralism in moderate environmental conditions during the early autumn, investigating possible associations with environmental temperature (T), humidity (H), and light intensity. Our hypothesis was partially supported by the data, as a positive association was found with the ambient temperature, but there was a negative relationship with the humidity. A strong positive correlation was also found between both ET and FT and ambient temperature, indicating that as the ambient temperature increases, both ET (0.7887) and FT (0.6280) increase significantly. This aligns with existing literature on camel thermoregulation, which highlights their ability to tolerate elevated body temperatures [26,27,49]. The data showed a strong negative correlation between humidity and temperature (−0.8785), with coefficients of −0.7444 for ET and −0.5519 for FT. This suggests that high humidity was associated with lower eye and flank temperatures, likely due to the reduced effectiveness of evaporative cooling mechanisms in humid conditions [25]. These results signifying that these camels were utilizing various physiological adaptations to cope with the heat, as suggested in the literature [25,50]. Understanding these relationships is vital for developing effective management strategies to mitigate heat stress in dromedary camels.

ET was not affected by age or sex; only by physiological status. The young camels showed higher ET values (35.41 ± 0.33), but this finding was not statistically significant (*p* < 0.353). This is because they likely need a longer time to adapt to their environment, as fatty pads are deposited in the face around the ocular region and throughout the body at a later stage. These fatty pads indeed assist in providing protection and thermal insulation for the eyes. The echogenic mass on the dorsal surface of the iris up to the dorsal pupillary margin functions as a *granula iridica* in equine eyes [51] for protecting the lens, retina, and choroid from direct exposure to sunlight [52]. This could be the main reason for the camels’ consistent ET ranges during this study. Air is retained within the layers of lipid-filled cells and the keratin matrix, such as in feathers and hair, thereby enhancing thermal insulation in animals and regulating their thermal conductivity [19]. The ET remained the same in the males and females, proving that both sexes manage their thermal stress in the same manner. Only the pregnant camels exhibited a lower ocular temperature, but this could have been due to their unique metabolic demands, hormonal status, and behavior, leading to higher thermal adaptability, as already reported in the literature [53].

ET was positively associated with the environmental temperature but negatively correlated with humidity, with an ET variation coefficient three times lower than FT. This can be a window for evaluating the animal’s core body temperature [28,54]. The trend of these ocular temperatures could be due to the existence of a local critical temperature (harmful for eye). At that critical range, the camel’s eye tissues may be damaged, thus compromising brain structure and function [14].

Any elevation in ET can indicate increased metabolic activity and potential overheating. The maxillary artery vascularizes the ocular region and aids in heat dissipation by maintaining a temperature ~0.5 °C lower than the brain [36]. This difference of a few degrees and consistency in ocular temperature demonstrates the role of the brain, mediated through the optic nerves, in facilitating communication between the orbit and the cranium. This interaction reflects the health of the eye [55,56]. These results indicate that ET may provide early indicators of thermal stress, allowing for timely interventions such as providing shade or water. The flank temperature in the dromedary camels remained relatively stable across the age classes, sexes, and physiological statuses, as evidenced by the high *p* values (0.5867, 0.7713, and 0.1163) associated with each category. This suggests that dromedary camels possess a sophisticated thermoregulatory system characterized by adaptive heterothermy, with eye and flank temperatures serving as key indicators of their thermal state. These adaptations enable them to thrive in arid environments by effectively managing body temperature fluctuations in response to both internal and external stimuli. The average FT values remained the same among the age groups. Our results have the same patterns of stable thermoregulatory responses as those shown in dromedary camels under various conditions [57,58,59]. This reinforces the notion that camel thermoregulation is robust, irrespective of their developmental stage. Meanwhile, factors such as environmental conditions and metabolic rates may have a more pronounced effect on FT [59]. The average FT for the males was 37.18 °C, while the females had an average FT of 36.83 °C, reflecting a statistically non-significant difference (0.7713). Our findings do not match research supporting that sex has a significant role in influencing the thermal parameters of dromedary camels due to various factors including hormones [57]. Concerning physiological status, the average FT varied among the following groups: breeding (37.42 °C), immature (37.06 °C), lactating (36.73 °C), non-lactating (37.89 °C), and pregnant (36.31 °C). The *p* value (0.1163) indicated no significant differences among these categories, suggesting that lactation and pregnancy do not substantially affect FT. These findings are mainly due to the camels’ adaptations for maintaining thermal homeostasis despite varying metabolic needs [26,60].

Skin is a complex architectural blend of tissue covering the body, assisting animals in thermoregulation and water balance. The flank fur and hair coat of camels play a crucial role in their thermoregulation. A camel’s thick coat consists of two layers, a dense undercoat and longer guard hairs, which together provide insulation against both heat and cold [61]. The flank has a thicker dermis and hypodermis compared to other areas, enhancing its ability to act as a thermal buffer [62]. During periods of high ambient temperature, camels can allow their body temperature to rise significantly, utilizing their fur to minimize heat gain from the environment. This adaptation reduces the temperature gradient between the body and external conditions, helping to conserve water by limiting evaporative cooling [25]. Moreover, the structure of the hair coat reflects sunlight and provides shade, further aiding in temperature regulation [63]. Overall, the combination of a thick fur coat and specific adaptations in the flank region enables camels to maintain a stable core body temperature while effectively managing external thermal challenges.

Also, the sweat glands in camels are unique and distributed in association with each primary hair follicle. This anatomical arrangement allows their ducts to open into the upper portion of the hair follicle at a specialized structure, which facilitates sweat secretion directly into the hair follicles. This mechanism helps to cool the skin surface while minimizing water loss; a vital adaptation for survival in arid climates [64].

Research has shown that dromedary camels can tolerate elevated body temperatures during thermal stress without significantly changing their thermoregulation [17]. The FTAvg in dromedary camels is significantly influenced by ambient temperature and humidity, exhibiting a pattern similar to that of ET. When exposed to high ambient temperatures, increased cutaneous blood flow facilitates heat distribution throughout the body, creating a temperature gradient between the inner and outer environments [65]. This may be due to the camel’s skin epidermal strata, which acts as a protective barrier between the body and the outside environment [21]. However, variations in FT are generally lower compared to ET, likely due to the insulating properties of camel fur [65].

The interaction between humidity and FT in camels is characterized by a moderate negative correlation. This relationship can be attributed to several physiological and environmental factors. Higher ambient temperatures enhance water evaporation from surfaces, which, upon entering the atmosphere, displaces existing air and reduces relative humidity (RH). This phenomenon aligns with psychrometric principles describing the interaction between temperature and humidity. As core body temperature increases without a corresponding rise in moisture levels, RH decreases, potentially leading to discomfort and heat stress [18].

Research indicates that livestock exposed to high ambient temperatures (above 35 °C) combined with low humidity levels (below 30%) exhibit signs of heat stress. Dromedary camels have a wider thermo-neutral zone (TNZ) at elevated ambient temperatures compared to other species due to unique morpho-physiological adaptations. The negative correlation between humidity and body temperature reflects the challenges posed by high humidity on evaporative cooling mechanisms. Increased humidity saturates the air with moisture, hindering effective thermoregulation through evaporation. This saturation can compromise adaptive heterothermy, which allows camels to manage diurnal temperature fluctuations of up to 6 °C while minimizing water loss through sweating [1]. Research has shown that the sweat glands of camels are of different size and shapes and are aggregated in large numbers spread in the dermis along with varied thicknesses of the layers [66].

ET and FT were not influenced by light intensity. This could be due to the coat color and the characteristics of the examined camels that are responsible for the absorbed heat and radiation [67,68] due to the epidermal strata. Camel coat characteristics include thickness, density, diameter, color, and texture, significantly impacting thermal insulance. Darker-colored camels are more prone to heat absorption than lighter-colored camels. This is in accordance with our data due to their better reflectivity of solar radiation [69,70]. Therefore, *Marecha* camels can easily survive in hotter climates and months due to thermoregulatory capacity [71]. Skin pigmentation helps in protecting the deep tissues against excess solar radiation [72]. Variations in hair length and color can affect the camel’s body temperature regulation by up to 2 °C [73]. The thick fur on camels’ flanks and legs, along with adipose stores concentrated in the dorsal hump, play a crucial role in thermal regulation [74]. During sweating, the longer hairs provide insulation without becoming saturated, thereby maintaining their insulating properties. This proves the role of the flank and auxiliary regions as thermal regulators [64].

Our findings need to be interpreted with caution, as there were some limitations. First of all, the distribution of the animals among the classes was not even, as there were fewer males due to the fact the males are usually sold for meat and their numbers are always lower in dairy camel herds. Moreover, we could not monitor and record the rectal temperature (RT) of the animals, as this is a very invasive procedure which requires restraint, and these animals were not often tamed. Consequently, we could not calculate any association between ET, FT, and RT. Finally, this study was conducted in moderate weather conditions, but the situations may change when the camels are outside their TNZ. Notwithstanding these limitations, this study has increased our knowledge of the possible use of IRT in monitoring heat stress in camels.

## 5. Conclusions

This study provides evidence that eye and flank temperatures recorded using IRT in dromedary camels are positively associated with environmental temperature and negatively associated with relative humidity. Our findings may indicate that higher humidity levels may hinder evaporative cooling mechanisms, leading to lower thermal readings in both body regions. While the lux data did not have any influence on the ET and FT. The camels’ flank region temperatures were not affected by age, physiological status, or sex, but the ET was significantly affected their physiological status, with pregnant camels exhibiting lower values. Our results are novel and could help in understanding the possible use of IRT in monitoring the welfare of dromedary camels.

## Figures and Tables

**Table 1:** Average eye temperature of dromedary camels with respect to age classes, sex, and physiological status.

Category		N	Average Eye Temp (°C) ± SE *	*p* Value *
**Age**	Adult	245	34.98 ± 0.11	0.3530
	Pubertal	229	35.14 ± 0.11	
	Young	25	35.41 ± 0.33	
**Sex**	Male	15	35.04 ± 0.43	0.9296
	Female	484	35.08 ± 0.07	
**Physiological Status**	Breeding male	5	34.78 ± 0.74 ^ab^	0.0458
	Immature female	10	35.17 ± 0.52 ^ab^	
	Lactating	227	35.20 ± 0.11 ^a^	
	Non-lactating	97	35.32 ± 0.17 ^a^	
	Pregnant	160	34.75 ± 0.13 ^b^	

* All pairwise comparisons were performed at 5% level of significance; ab means in the same row with different superscript letters are different at *p* ≤ 0.05.

**Table 2:** Average flank temperature (FT) of dromedary camels with respect to age classes, sex, and physiological status.

Category		N	Average Flank Temp (°C) ± SE	*p* Value
**Age**	Adult	241	36.62 ± 0.30	0.5867
	Pubertal	226	37.03 ± 0.31	
	Young	27	37.12 ± 0.88	
**Sex**	Male	15	37.18 ± 1.19	0.7713
	Female	479	36.83 ± 0.21	
**Physiological Status**	Breeding	5	37.42 ± 2.05	0.1163
	Immature	10	37.06 ± 1.45	
	Lactating	226	36.73 ± 0.30	
	Non-lactating	97	37.89 ± 0.46	
	Pregnant	156	36.31 ± 0.37	

**Table 3:** Association of the average eye and flank temperatures of dromedary camels with their environmental temperature, relative humidity, and lux.

Independent Variables		Regression	R^2^ Adjusted	Tolerance I-R^2^	VIF * (1/Tolerance)
**Eye**	Temperature	ETA = 24.7476 + 0.3092T	0.6399	0.3594	4.4
	Humidity	ETA = 38.5857 − 0.0727H	0.4938	**0.5052**	**4.4**
	Lux	ETA = 35.20 + 0.000001678Lux	0.0024	**0.9948**	**1.0**
**Flank**	Temperature	FTA = 13.4236 + 0.7012T	0.4299	0.5689	4.4
	Humidity	FTA = 44.3975 − 0.1564H	0.2999	**0.6987**	**4.4**
	Lux	FTA = 36.71 + 0.0000426Lux	0.0445	**0.9529**	**1.0**

* One part is about descriptive statistics of average eye temperature and average flank temperature, and second part is a regression analysis; the software used was statistix8.1. * Interpretation: VIF  ≥  5 (highly correlated); 1  <  VIF  <  5 (moderately correlated); VIF  >  1 (not correlated).

**Table 4:** Pearson correlations between average Eye Temperature (ET) and average Flank Temperature (FT) with relative humidity and ambient temperature.

Sr. No.	Category	r	Remarks
1	ET and FT	0.6643 ***	If eye temperature ↑ then flank temperature also ↑
2	T and H	−0.8785 ***	If temperature ↑ then humidity ↓
3	H and ET	−0.7444 ***	If humidity ↑ then eye temperature ↓
4	H and FT	−0.5519 ***	If humidity ↑ then flank temperature ↓
5	T and ET	0.7887 ***	If temperature ↑ then eye temperature also ↑
6	T and FT	0.6280 ***	If temperature ↑ then flank temperature also ↑
7	Lux and ET	0.1019 *	If lux ↑ then eye temperature also ↑
8	Lux and FT	0.2650 ***	If lux ↑ then flank temperature also ↑
9	Lux and H	−0.0579 ^NS^	If lux ↑ then humidity ↓
10	Lux and T	0.0674 ^NS^	If lux ↑ then temperature also ↑

* Indicates statistical significance (*p* < 0.05); *** indicates statistical significance (*p* < 0.001), meaning the correlations are unlikely to be due to chance. ↑ = increase; ↓ = decrease. NS: Non statistically significant.

## Data Availability

The data presented in this study are available from the corresponding author upon reasonable request.
